# Monitoring the Effect of Transdermal Drug Delivery Patches on the Skin Using Terahertz Sensing

**DOI:** 10.3390/pharmaceutics13122052

**Published:** 2021-12-01

**Authors:** Hannah Lindley-Hatcher, Jiarui Wang, Arturo I. Hernandez-Serrano, Joseph Hardwicke, Gabit Nurumbetov, David M. Haddleton, Emma Pickwell-MacPherson

**Affiliations:** 1 Department of Physics, University of Warwick, Coventry CV4 7AL, UK; Hannah.Hatcher@warwick.ac.uk (H.L.-H.); Arturo.Hernandez-Serrano@warwick.ac.uk (A.I.H.-S.); 2 Department of Electronic Engineering, The Chinese University of Hong Kong, Hong Kong 999077, China; wangjr3399@outlook.com; 3 Department of Plastic Surgery, University Hospitals of Coventry and Warwickshire NHS Trust, Clifford Bridge Road, Coventry CV2 2DX, UK; J.Hardwicke@warwick.ac.uk; 4 Warwick Medical School, University of Warwick, Coventry CV4 7HL, UK; 5 Medherant Ltd., The Venture Centre, University of Warwick Science Park, Coventry CV4 7EZ, UK; g.nurumbetov@medherant.co.uk; 6 Department of Chemistry, University of Warwick, Coventry CV4 7AL, UK; D.M.Haddleton@warwick.ac.uk

**Keywords:** THz time domain spectroscopy, transdermal drug delivery, occlusion, skin hydration

## Abstract

Water content of the skin is an important parameter for controlling the penetration rate of chemicals through the skin barrier; therefore, for transdermal patches designed for drug delivery to be successful, the effects of the patches on the water content of the skin must be understood. Terahertz (THz) spectroscopy is a technique which is being increasingly investigated for biomedical applications due to its high sensitivity to water content and non-ionizing nature. In this study, we used THz measurements of the skin (in vivo) to observe the effect of partially and fully occlusive skin patches on the THz response of the skin after the patches had been applied for 24 h. We were able to observe an increase in the water content of the skin following the application of the patches and to identify that the skin remained hyper-hydrated for four hours after the removal of the fully occlusive patches. Herein, we show that THz spectroscopy has potential for increasing the understanding of how transdermal patches affect the skin, how long the skin takes to recover following patch removal, and what implications these factors might have for how transdermal drug patches are designed and used.

## 1. Introduction

Transdermal drug delivery is used for an increasing number of applications to provide alternatives to oral and intravenous administration, with over one billion patches being manufactured each year [1]. Some of the earliest treatments delivered through transdermal patches include the prevention of motion sickness [2,3], and nicotine to aid smoking cessation [4]. Patches have also been developed for hormone replacement therapy [5], localised pain relief using diclofenac [6] or fentanyl [7], and for controlling the symptoms of chronic conditions such as Parkinson’s [8] or Alzheimer’s disease [9].

Administration of drugs through the skin can be a useful option to avoid potential side effects caused by metabolism of the drug in the liver or gastrointestinal tract and to avoid the use of needle penetration. It can also allow for the controlled release of a drug into the body at a more consistent rate than orally administered drugs over the entire time window of drug requirement [10]. Transdermal patches can be very beneficial in cases where the patient may lack the capacity to take medication by themselves, as the patches can be easily applied and replaced by a carer and can deliver the required dosage for a longer period than oral medication. A primary factor which can influence the rate of drug delivery through the patch is the hydration of the skin. It is therefore important that the effect of transdermal patches on the skin is understood as fully as possible [11]. Additionally, the way in which the skin recovers following the removal of the patches is important as it can inform the advice given to patients about how often to apply patches and how often the application site should be rotated.

There are many aspects to be considered when designing patches for transdermal drug delivery including the choice of adhesive and backing material. Changing the backing material can alter the occlusive nature of the patch and change how the skin responds to the application of the patch. In some cases, such as with fentanyl patches, where the drug is highly potent and potentially toxic if administered to the wrong person, it is important that a fully occlusive backing is used to prevent the leaking and transfer of the drug [12]. However, it has also been observed that the long-term application of fully occlusive patches can cause skin irritation due to the buildup of water at the surface of the skin [13].

Some studies have been performed to assess the effects of occlusion on the water content of the skin and the function of the skin barrier. For example, Aly et al. observed an increase in the trans-epidermal water loss (TEWL) measured following 24 h of occlusion with plastic [14]. They then observed that the TEWL had returned to its previous levels 18 h after the removal of the plastic, suggesting that the occlusion of the skin had increased the levels of water in the skin but that there had been no significant damage to the skin barrier. A subsequent study by Faergemann et al. measured an increase in the water content of the skin with a dielectric probe following occlusion with a plastic layer [15]. An increase in the bacteria count was also observed with increasing occlusion time, suggesting that in some cases long term occlusion of the skin can increase the risk of bacterial infections.

Terahertz (THz) light is in the range of electromagnetic waves with frequencies between 100 GHz and 10 THz. With the increasing availability of commercial THz generation systems there has been rapid expansion in the exploration of potential applications of THz spectroscopy in recent years. In particular, there is interest in biomedical applications which can take advantage of the strong sensitivity of THz light to water content in biological tissues and other associated changes including the presence of cancerous tissue. The low photon energy (0.4–40 meV) of THz light means that it is non-ionizing, making THz light particularly attractive for in vivo applications. Groups have identified contrasts between healthy and cancerous tissue with many types of cancer including skin cancer [16], breast cancer [17], gastric cancer [18] and oral cancer [19], among others using both in vivo and ex vivo studies. Additionally, there have been investigations into other potential clinical applications of THz measurements including corneal assessments [20] and the diagnosis of diabetic foot syndrome [21]. Studies have also demonstrated the ability of THz spectroscopy and imaging techniques to quantitatively assess the severity and depth of burns [22,23].

Previous studies have found that the strong sensitivity of THz light to the water content of skin can also be used to observe changes induced by occlusive materials which prevent the natural loss of water through the skin barrier. This was identified as a potential problem when performing THz measurements which involve contact between the skin and an imaging window, as the imaging window has an occlusive effect on the skin, meaning that the water profile in the skin changes throughout the measurement [24]. These undesirable effects can be removed by numerical methods. However, Wang et al. demonstrated that this sensitivity to the effects of occlusion on the skin can also be a strength [25]. They successfully used THz measurements to observe changes in the skin following the application of silicone gel sheeting, which is commonly used to treat severe burns by occluding the skin and increasing the water content. Additionally, Sun et al. found that by using a layered model with effective medium theories, the diffusivity of the skin under occlusion could be extracted with THz measurements [26].

THz spectroscopy and imaging techniques have previously been applied to assess the efficacy of various approaches to transdermal drug delivery. For example, Kim et al. were able to measure the penetration of a sample containing ketoprofen through excised mouse skin by taking THz images of the underside of the skin sample [27]. Wang et al. then investigated the effects of approaches to enhance the penetration of the samples through the skin barrier, such as micro and nano needle patches. They performed ex vivo measurements using porcine skin and observed that there was a significant change in the measured THz response of a sample that was treated with a nanoneedle patch prior to drug application compared to skin which received no extra treatment before the drug was applied [28]. THz measurements have also been used to quantify the amount of nicotine that has left a nicotine patch by measuring the patch before and after it was applied for different durations, showing that it is possible to use THz spectroscopy to verify the rate of drug delivery through a patch membrane [29]. These studies focused on the penetration of the drug through the skin, whereas in this paper we investigated the effect of patches on the skin in order to learn how different patches can influence the rate of drug delivery through the skin barrier. However, it is possible that in the future the approach highlighted in this paper could be combined with those mentioned above to use THz spectroscopy and imaging to obtain a more complete picture of the dynamic response of the skin to the application of patches containing active drug ingredients.

In this study, we investigated the ability of in vivo THz measurements of the skin to identify the changes induced in the skin by patches for transdermal drug delivery by analyzing THz light reflected from the surface of the skin. We tested two types of patches: one backed with a polyethylene terephthalate (PET) film which is fully occlusive and the other backed with a woven material which is semi-occlusive. Three percentages of excipients were tested for each type of patch and the properties of the skin were measured immediately after the removal of the patches following application for 24 h. Additionally, the recovery of the skin was monitored by taking subsequent measurements 30 min and four hours after the removal of the patches. We used in vivo THz measurements to identify changes in the water content of skin treated with different types of patches and observed the changes further induced in the skin when it was occluded by a quartz imaging window during the measurement following the removal of the patches.

## 2. Materials and Methods

### 2.1. Skin Patches for Assessment

**Figure 1:**
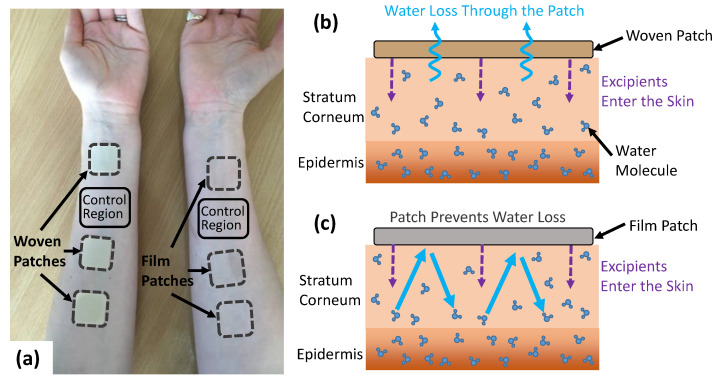
(**a**) The volar forearms of a subject with the woven- and film-backed patches applied with a control region left untreated on each arm. (**b**,**c**) show the mechanism by which the woven- and film-backed patches affect the movement of water in the skin.

In this study, we tested the effects of drug in adhesive patches for transdermal drug delivery on the THz response of the skin. To isolate the effects of the patch from the effects of the drugs, the active ingredient was not present in these patches. Two different types of backing materials were used to investigate the effect of the occlusive nature of the patch backing on the THz response of skin, a fully occlusive PET film backing and a partially occlusive fabric woven PET fibre backing. An example of the patches applied to the volar forearm can be seen in Figure 1a. It was expected that the woven-backed patch would allow some water loss from the skin through the adhesive and out from the backing, whilst the PET film-backed patch would prevent all water loss from the skin surface as it is impermeable to water vapour, as shown in Figure 1b,c. Additionally, three excipient compositions were tested with 0%, 3% and 6% propylene glycol, which is commonly added to transdermal drug patches to enhance the penetration of drugs through the skin barrier. These concentrations of propylene glycol were chosen to replicate the concentrations typically used in a transdermal drug patch, making it possible to investigate the potential effects of these different adhesive compositions on the hydration of the skin. The patches were manufactured by Medherant Ltd. with varying propylene glycol contents present in the adhesive. Additionally, 10% transcutol was present in the adhesive of all the patches [30]. Both propylene glycol and transcutol are FDA approved excipients for transdermal and topical medicines and are used to increase the diffusion of the drug across the skin. Frequently, the effects of these approved excipients vary from the active pharmaceutical ingredient (API) and have cooperative effects. The adhesive used was a proprietary polyurea cross-linked thermoset mainly composed of a high percentage weight of siloxy-terminated poly (propylene glycol) prepolymer chain extended with isophorone diisocyanate and cross-linked and cured with the excipients in place by exposure to 100% humidity for five minutes at 70 °C [31,32].

### 2.2. Terahertz Measurements of the Skin Using a Reflection Geometry

**Figure 2:**
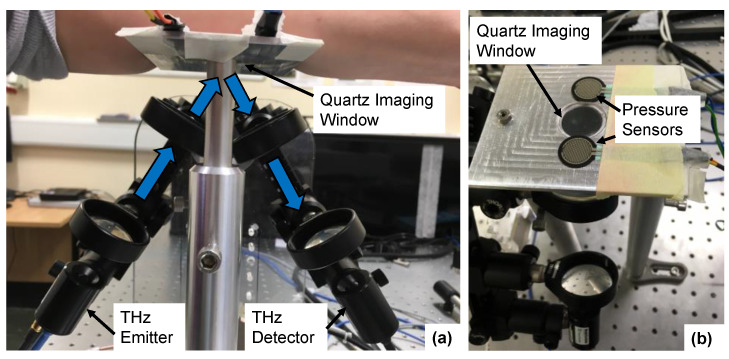
The experimental setup used to take THz measurements of the skin with a reflection geometry, from the front (**a**) showing the THz emitter/detector and from above (**b**) showing the pressure sensors positioned either side of the quartz imaging window.

The THz measurements of the skin were acquired with a fibre-coupled TeraSmart THz spectrometer from Menlo systems GmbH (Planegg, Germany). This THz time-domain spectroscopy (TDS) system can deliver broadband electromagnetic pulses with a temporal duration of 1 ps with a usable bandwidth ranging from 0.1 to 5 THz. In this study, we used results in the frequency range from 0.1 to 1 THz, as higher frequencies are more sensitive to the effects of scattering from the roughness of the skin surface. The THz emitter and detector were mounted on optical rails at an angle of 30 degrees to the quartz imaging window, as shown in Figure 2. The system can acquire THz pulses at a rate of 4 pulses per second. In each skin measurement, 240 pulses were measured, meaning that the skin was in contact with the imaging window for one minute at each time point. This relatively long measurement duration was chosen in order to measure the response of the skin to the occlusive effects of the imaging window and to observe how this changed following application of the different types of patches.

### 2.3. Protocol for Skin Measurements

To test the effect of the patches on the skin, three PET fibre woven-backed patches and three PET film-backed patches were applied to the volar forearms of five human subjects. The subjects involved in this study were four males and one female in the age range 24–34. All the subjects had healthy skin in the regions in which the measurements were performed. Written informed consent was obtained from each subject prior to their involvement in the study. The patches were applied for 24 h before being removed and then the skin was assessed. THz measurements were performed on each region prior to the application of the patch, in addition to a control region on both forearms which remained untreated throughout the study. The regions were subsequently measured with THz light immediately following the removal of each patch and 30 min and four hours after patch removal and the change in the reflected light was measured in order to observe the recovery of the skin. The control regions were also measured at each time point. These time points were chosen in order to assess the changes in the water content of skin caused by the application of the patches, in addition to the short- and long-term recovery of the properties of the skin following the removal of the different types of patches.

To ensure that the results were as repeatable as possible, the measurements were performed according to the robust protocol for THz measurements of the skin previously outlined by Lindley-Hatcher et al. [33]. As part of this, the subjects were asked to spend 30 min in the laboratory prior to each measurement, to give the skin time to acclimatise to the controlled environmental conditions of the laboratory. Additionally, previous studies have found that the applied pressure between the skin and the imaging window can affect the measured THz response of skin [34]. Therefore, pressure sensors were positioned either side of the quartz imaging window, as shown in Figure 2; this was part of a pressure sensor system which is described in more detail in reference [33]. The pressure sensor was connected directly to the THz system to provide a real time output which was visible to the participants to help them maintain the applied pressure within the desired range. The measured output from the pressure sensors was also recorded so that these values could be used to help with the processing of the results, making it possible to reject measurements acquired at the incorrect pressure. The target pressure range defined for this study was 1.6–2.0 Ncm^−2^. This was found to be a large enough pressure to reduce the risk of air gaps being present between the skin and the imaging window during the measurements, whilst being low enough that it could be comfortably maintained by the subject throughout the measurement [33]. The pressure sensors were also used to start the THz measurement—as soon as the pressure sensors detected any contact the measurement began. This ensured that the time for which the skin was in contact with the imaging window was controlled for all subjects.

### 2.4. Approach to Data Analysis

**Figure 3:**
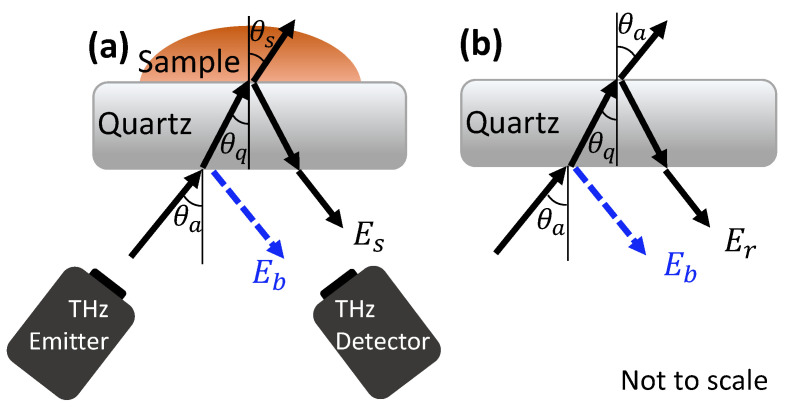
The reflection of an incident THz pulse from the quartz imaging window when the sample, $E_{s}$ (**a**) and reference, $E_{r}$(**b**) measurements are acquired. The baseline reflections, $E_{b}$, from the underside of the quartz imaging window are shown in blue.

THz measurements of the sample were acquired in a reflection geometry with the skin in contact with a quartz imaging window. Some portion of the THz pulse was reflected at each interface where there was a difference in the THz properties of the two layers, for example at the interface between air and the imaging window, and the interface between the imaging window and skin, as shown in Figure 3a. When the measurements were acquired two pulses were therefore observed—one from the quartz-skin interface, $E_{s}$ and one from the air-quartz interface on the underside of the imaging window, $E_{b}$. To identify the properties of the sample, this reflection from the underside of the imaging window (used for calibration) must be removed using an approach called baseline subtraction; this is described fully in the work by Chen et al. [35]. Once the sample pulse had been isolated, it was then Fourier transformed to become dependent on frequency, ω, and processed using the approach defined in Equation (1) where $E_{r}\left(\omega \right)$ is the Fourier transform of the reference pulse which is a measurement of the quartz window without the sample, as shown in Figure 3b. A double Gaussian filter was also applied to remove noise [24].

(1) $$processed\;signal=iFFT\left(FFT\left(filter\right)\times \frac{E_{s}\left(\omega \right)}{E_{r}\left(\omega \right)}\right)\;$$

**Figure 4:**
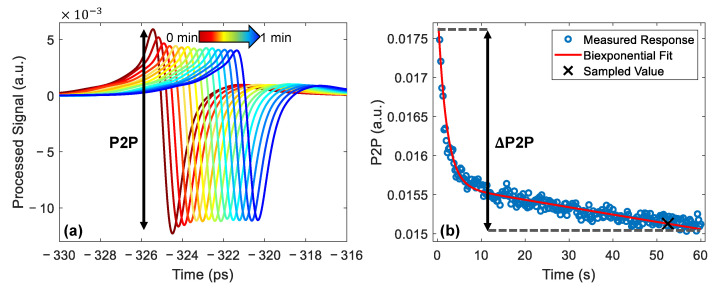
(**a**) The processed THz signals measured from the untreated skin of a single subject, the colour gradient shows increasing occlusion time. The pulses are plotted every second for the first three seconds then every five; they have been shifted horizontally for clarity. The definition of the peak-to-peak (P2P) variable is shown on the plot. (**b**) The P2P plotted as a function of occlusion time, fitted with a bi-exponential function shown by the red line and sampled 52 s into occlusion, as shown by the black cross. The definition of the ΔP2P variable is shown as the difference between the value of the fit at the start and end of occlusion.

Examples of the processed signal are shown in Figure 4a. In this figure, the signals are plotted every second for the first three seconds of the measurement and every five seconds for the remainder of the one-minute measurement. The pulses are shifted horizontally in the figure for clarity. In this figure, the peak-to-peak (P2P) variable is defined as the amplitude of this processed signal; this can be used to measure changes in the THz response of the skin.

The P2P decreased throughout the one-minute measurement, as shown in Figure 4a, as the imaging window had an occlusive effect, causing water to build up in the surface of the skin, reducing the reflection strength at the quartz-skin interface. The decrease in the P2P was associated with an increase in the water content and monitoring changes in the P2P made it possible to quantify changes in the water content of skin caused by the application of the patches. Sun et al. showed that the decrease in the P2P as a function of occlusion time can be modelled using a bi-exponential function [24]. For each measurement, the results recorded by the pressure sensor were used to remove P2P values which were recorded when the incorrect pressure was being applied. After the measurements were filtered to select only the values obtained at the correct pressure the bi-exponential fitting was applied and the value of the fit was sampled 52 s into occlusion, as shown in Figure 4b, using the approach we introduced in our previous work [36].

The application of fully or partially occlusive transdermal drug patches changes the distribution of water in the skin; this changes the way in which the skin will respond to occlusion by the imaging window. In order to quantify this change in the response to occlusion, we defined the variable ΔP2P as the difference between the value of the bi-exponential fit at the start and end of occlusion, as shown in Figure 4b. This variable could then be used to observe whether the application of the patches increased or decreased the gradient of the occlusion curve observed throughout the one-minute measurement.

In addition to using the amplitude of the time domain signal, it is possible to extract the frequency dependent optical properties of the sample, such as the refractive index and absorption coefficient. The frequency dependent sample to reference ratio $E_{s}\left(\omega \right)/E_{r}\left(\omega \right)$ was used to obtain the complex refractive index $\tilde{n},$ using Equations (2) and (3) which use the Fresnel equations and Snell’s law for s-polarised incident waves [35]:

(2) $$\frac{E_{s}\left(\omega \right)}{E_{r}\left(\omega \right)}=\;\frac{r_{qs}}{r_{qa}}=\frac{{\tilde{n}}_{q}\cos\theta_{q}-{\tilde{n}}_{s}\cos\theta_{s}}{{\tilde{n}}_{q}\cos\theta_{q}+{\tilde{n}}_{s}\cos\theta_{s}}\times \frac{{\tilde{n}}_{q}\cos\theta_{q}+{\tilde{n}}_{a}\cos\theta_{a}}{{\tilde{n}}_{q}\cos\theta_{q}-{\tilde{n}}_{a}\cos\theta_{a}},$$

(3) $${\tilde{n}}_{a}\sin\theta_{a}={\tilde{n}}_{q}\sin\theta_{q}={\tilde{n}}_{s}\sin\theta_{s},$$

where $r_{qs}$ and $r_{qa}$ are the Fresnel coefficients for the reflection at a quartz-skin and quartz-air interface, respectively, and ${\tilde{n}}_{q}$,$\;{\tilde{n}}_{s}$, ${\tilde{n}}_{a}$, $\theta_{q}$, $\theta_{s}$ and $\theta_{a}$ are the complex refractive indices and incident angles for quartz, skin and air, respectively, as indicated in Figure 3. The complex refractive index is defined as: $\tilde{n}=n-i\kappa ,$ where n is the real part of the refractive index (referred to simply as the refractive index throughout the rest of this paper), and κ is the extinction coefficient. The extinction coefficient can be used to extract the absorption coefficient α using the relation: α=2ωκ/c, where ω is the frequency and c is the speed of light.

In previous studies, we introduced a variable called the normalised relative change (NRC) which can be used to remove the effect of natural variation of the skin between measurements which is not caused by the treatment of the skin [33]. In this study, the use of the NRC to characterise the changes observed in the skin is particularly important due to the relatively long time for which the patches were applied, giving the skin time to change significantly due to exposure to other environmental factors. The NRC is defined,

(4) $$\mathrm{NRC}\;\left(\%\right)=\frac{\left(X_{Tt}-X_{T0}\right)-\left(X_{CT}-X_{C0}\right)}{X_{T0}+\left(X_{CT}-X_{C0}\right)}\times 100,$$

where $X_{Tt}$ and $X_{Ct}$ are the THz responses measured at time *t* in the treated region and control region, respectively, and $X_{T0}$ and $X_{C0}$ are the values measured at time *t = 0* (before the patches were applied) in the treated and control regions.

The one-way ANOVA (analysis of variance) test was used to evaluate if the changes observed were statistically significant. These results were analysed with the Tukey–Kramer test to ensure that the threshold of 5% significance was maintained. These results can be used to see if the changes observed in the skin following the application of the patches were significant and to see if the patches produced significantly different results from one another.

## 3. Results

### 3.1. Average Changes in the Skin following Patch Application

**Figure 5:**
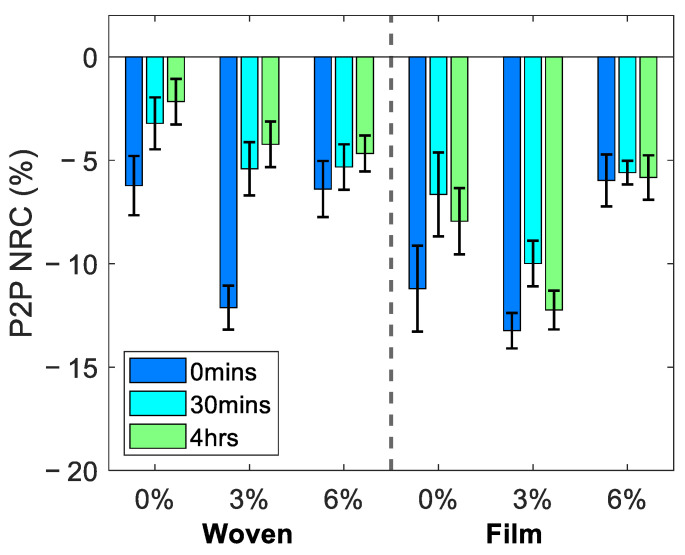
The average normalised relative change (NRC) in the P2P sampled 52 s into occlusion, observed in all five subjects at 0 min, 30 min and four hours following the removal of the woven- and film-backed patches containing 0%, 3% and 6% propylene glycol. The error bars are the standard error on the mean.

The average change in the reflected THz signal following the removal of each of the patches is shown in Figure 5, where the error bars indicate the standard error of the mean. The figure shows the NRC as defined in Equation (4) in the P2P amplitude of the THz pulse, sampled by fitting the occlusion curve with a bi-exponential function and sampling the value from the fit 52 s into occlusion. We associate a decrease in the P2P with an increase in the water content of the skin. The left- and right-hand sides of the figure show the responses observed following treatment with the woven- and film-backed patches respectively for the three excipient levels contained in the patches. The recovery of the skin following the removal of the patches can be observed from the results showing the measured properties of the skin 30 min and four hours after the patch was removed, in addition to the measurements taken immediately following patch removal.

For all the patches a decrease in the P2P was observed suggesting that the hydration levels of the skin had increased following application of each of the patches for 24 h. These changes appeared to persist in the skin for at least four hours following the removal of the patches. The results show that the film-backed patches induced a larger change in the skin than the woven patches and that the rate of recovery of the skin treated with the PET film-backed patches was reduced compared to the partially occlusive woven patches. Additionally, patches with 3% excipient levels seemed to have the largest impact on the THz response of the skin, however measurements on more subjects will be needed to draw further conclusions about the significance of this observation.

### 3.2. Effect of Patch Application on the Response of Skin to Occlusion

In addition to sampling the value of the P2P measured at a particular time point using a bi-exponential function, as shown in Figure 4, it is possible to use the observed occlusion curve to learn about the changes in the water distribution in the skin caused by the application of the patches. It is shown in Figure 4 that in untreated skin it was possible to observe a decrease in the THz P2P throughout a skin measurement due to the occlusive effect of the imaging window. However, if the water content in the skin had been disrupted, we would expect to have been able to observe this as a change in the way the skin responded to the one minute of occlusion throughout the THz measurement.

**Figure 6:**
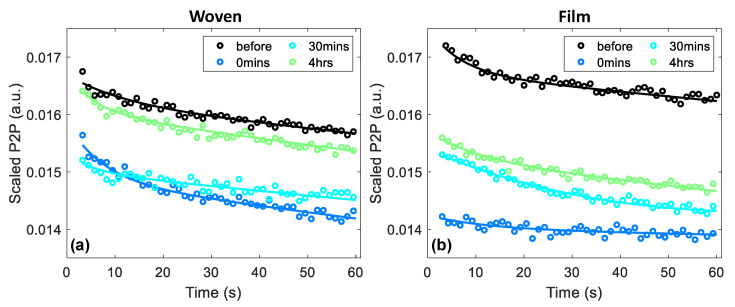
An example of the occlusion curves observed for a single subject following the application of (**a**) woven-backed and (**b**) film-backed patches; every fifth data point was plotted and the bi-exponential fits are shown by the solid lines. The results have been scaled by subtracting the change in the control region between the initial measurement and the measurement taken at that time point.

Figure 6 shows an example of the occlusion curves observed in a single subject following the application of a woven- and film-backed patch by plotting the change in the measured P2P throughout the one-minute measurement. Every fifth data point was plotted and the bi-exponential fit for the data set is shown by the solid lines. All these curves have been scaled to account for the natural variation of the skin between measurements. This was done by subtracting the difference in the control region between the initial measurement and the measurement taken at that time point, as sampled after one minute of occlusion. The black curves in each plot show the measured response of the region prior to the application of the patches. The coloured curves show the response of the skin to occlusion 0 min, 30 min and four hours after the patches were removed.

The dark blue curve in Figure 6a, which indicates the occlusion curve measured immediately following the removal of the woven-backed patch, appears to show a greater change in the P2P than that observed prior to patch application. Whereas the dark blue curve in Figure 6b, indicating the response following the removal of the film-backed patch, is much flatter than the response of untreated skin. The flattening of the occlusion curve suggests that the skin was already saturated with water due to the occlusive effect of the film-backed patch, so occlusion by the imaging window had little effect on the hydration of the skin. The steepening of the occlusion curve following removal of the woven-backed patch is harder to explain without further modelling of the skin.

At time intervals of 30 min and four hours after the removal of both patches, the behavior of the skin in response to occlusion by the imaging window had returned to what it was before the patches were applied as the slopes returned to the same shape. However, the P2P values remained lower than those of untreated skin, suggesting that there was an increase in the water content of the skin. The recovery of the skin with increasing time after the patches were removed can be seen as the occlusion curves rise back up towards the black curves measured before the patches were applied. It appears that the skin took longer to recover from the application of the film-backed patches than the woven-backed patches.

**Figure 7:**
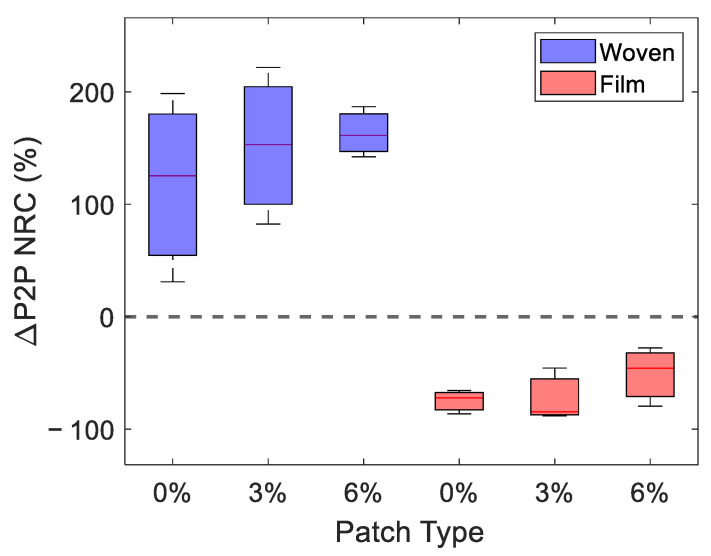
A box plot of the NRC in ΔP2P, a variable defined to give an indication of the change induced in the skin by one minute of occlusion by the quartz imaging window immediately after the removal of the patches. This figure shows the results in the three subjects who were able to keep the applied pressure within the desired range for the most time points. The red lines inside the boxes indicate the median response observed, while the upper and lower edges of the boxes show the upper and lower quartiles of the measured responses.

In order to visualise the change in the response to occlusion, Figure 7 shows the NRC of ΔP2P (as defined in Figure 4b). A positive NRC in ΔP2P indicates that the application of the patch increased the amount the P2P changed during one minute of occlusion and that the occlusion curve steepened. This approach provides a means of quantifying the qualitative changes observed in the occlusion curves, shown in Figure 6. In the boxplot, shown in Figure 7, the blue and red boxes show the response to the woven- and film-backed patches, respectively, where the upper and lower limits of the boxes indicate the upper and lower quartiles of the data set and the red lines through the boxes show the median. To produce this plot, we selected the three subjects who were able to keep the applied pressure within the desired range for the most time points. It is necessary to only study the subjects with the most consistent pressure to investigate ΔP2P as the occlusion curve is very sensitive to changes in pressure and any bumps in the data caused by movement of the subject or incorrect pressure are harder to account for.

We see a clear divide between the effects of the woven- and film-backed patches in Figure 7. The woven-backed patches appeared to increase the change in the skin under one minute of occlusion while the film-backed patches decreased this change. This confirms the qualitative trend observed in Figure 6. It does not appear that the percentage of propylene glycol excipient in the patch affected ΔP2P significantly. It seems that the dominant factor which influenced the way skin responded to occlusion was whether the patch applied was fully or partially occlusive—this would have determined whether the surface layers of the skin were already saturated with water prior to the occlusion of the skin by an imaging window.

### 3.3. Changes in Optical Properties

**Figure 8:**
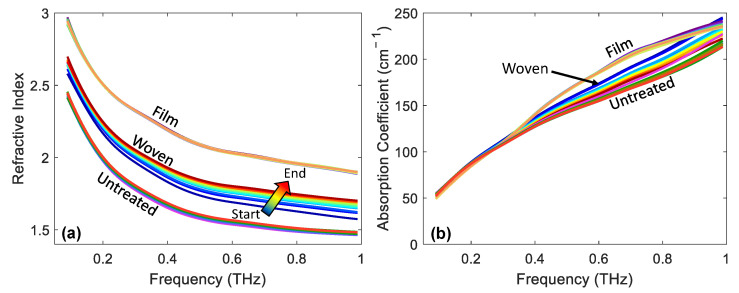
An example of (**a**) the refractive index and (**b**) the absorption coefficient as a function of frequency for untreated skin and immediately following the removal of film- and woven-backed patches. The colour gradients indicate increasing occlusion time, making it possible to observe the effect of the changing response of the skin to occlusion on the optical properties of skin.

Up until this point, all the results presented have involved analysis of how the P2P changed following the application of the patches. However, it is also possible to extract the frequency-dependent optical properties using the approach described in Section 2.4. Figure 8 shows the refractive index and absorption coefficient of the skin of a single subject for untreated skin and skin immediately following the removal of film- and woven-backed patches. The colour gradient indicates measurements being plotted later into occlusion with the coloured arrow showing the direction of increasing occlusion time and the lines are plotted every five seconds into occlusion.

It is clear from Figure 8a that there was a large difference in the refractive index of untreated skin and skin which had a film- or woven-backed patch applied. The application of the patches increased the refractive index suggesting that the water content of the skin had increased due to the patch application. The film-backed patch had the largest impact on the refractive index, suggesting that it caused the largest increase in the water content of the skin. Looking at the width of the distribution of the lines plotted with the colour gradient, it is possible to observe how much the refractive index of the skin changed throughout the one minute of occlusion by the quartz imaging window throughout the THz measurement. The largest distribution was shown by the skin which was treated with the woven-backed patch; this matched the trend observed in the time-dependent variable. This figure shows that throughout occlusion the refractive index of skin increased across the whole frequency range from 0.1–1 THz; this suggests that water built up in the surface layers of the skin. The smallest distribution of lines corresponding to the smallest change in the refractive index of the skin during occlusion was shown by the film-backed patches, confirming that the skin was already saturated with water so did not change further in response to further occlusion by the imaging window.

For completeness, we show the same results for the absorption coefficient of the skin in Figure 8b. The differences between the untreated skin and the skin treated with the different patches is less clear in this plot. There is some separation between the different treatments at higher frequencies, but we cannot learn as much about the effect of the patches on the skin from this plot.

### 3.4. Statistical Analysis

**Figure 9:**
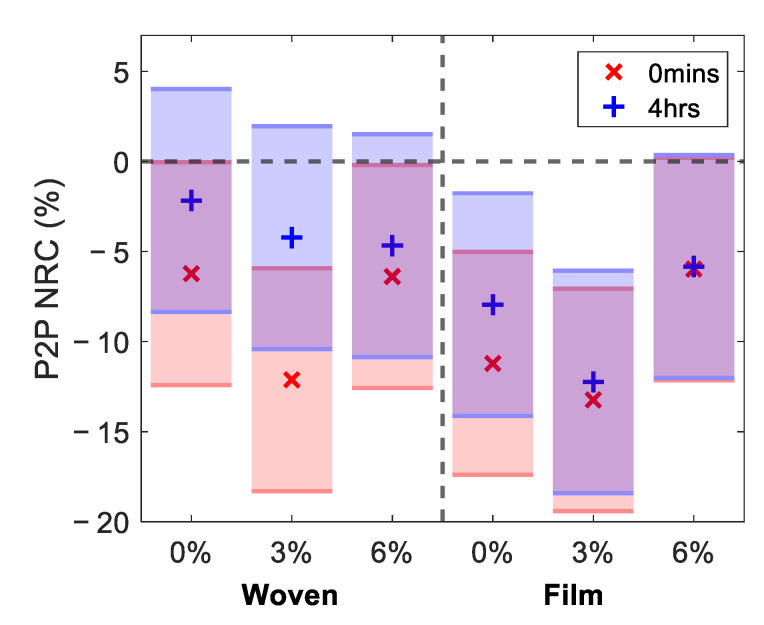
The results of performing the one-way analysis of variance test on the NRC in the P2P sampled 52 s into occlusion for all subjects; the markers show the estimated mean of the distribution of responses to the patches. The shaded bars are the 95% confidence intervals calculated using the Tukey–Kramer test.

Finally, we tested the statistical significance of the changes observed in the skin using the one-way ANOVA test and the Tukey–Kramer test [37]. The results of these statistical tests are shown in Figure 9, for the NRC in the P2P measured 0 min and four hours after the removal of the patches, where the shaded bars indicate Tukey’s minimal significant difference at a 5% significance level. Where the bars do not overlap with the dashed line indicating zero change, this indicates that there was a significant difference in the skin following treatment with the patches. The markers inside the bars indicate the estimated mean of the distribution of changes in the skin induced by each patch.

Studying the red regions in Figure 9, which indicate the results immediately after the patches were removed, reveals that all patches resulted in a significant change in the P2P. The patches with 3% propylene glycol led to the largest changes in the measured response of the skin for both patch types. The film-backed patches induced a larger change in the skin than the woven-backed patches. The blue regions show the results four hours after the patches were removed. These show that the changes in all regions of the skin treated with woven-backed patches were no longer significant, as all blue regions on the left-hand side of the plot overlap with the dashed zero line. The skin treated with the 0% and 3% propylene glycol patches backed with the PET film maintained a significant difference and the result for the 6% film-backed patch just touches the zero line. This confirms that the changes induced in the skin by the film-backed patches persisted for much longer than those induced by the woven-backed patches. From the measurements of the five subjects performed in this study, significant changes in the skin following the application of the two types of patches could be observed. However, in order to assess subtler changes in the skin, such as the statistical significance of the results for patches with different propylene glycol concentrations, a study involving more subjects should be performed.

## 4. Discussion

In this study we found that THz reflection measurements can be used to observe changes in the skin induced by the application of transdermal drug patches. It was possible to observe these changes in the time and frequency dependent properties, particularly in the amplitude of the processed THz signal reflected from the surface of the skin and the refractive index of the skin in the frequency range from 0.1 to 1 THz. The measured responses suggest that the 24 h application of the different patches all increased the water content of the skin.

The ability of the THz-TDS system used in this study to take rapid, repeated measurements of the skin whilst it remains in contact with an imaging window makes it possible to learn more about the effect of patch application on the distribution of water in the upper layers of the skin. The flattening of the occlusion curve observed in skin which had been treated with a film-backed patch suggests that the PET film fully occluded the skin, preventing the natural loss of water, causing the water levels in the skin to increase. Therefore, when the skin was occluded by the imaging window in order to perform the THz measurements there was little change observed in the THz response of the skin as the upper layers of the skin were already saturated with water. Surprisingly, we also observed a change in the response of skin treated with a woven-backed patch to occlusion as the occlusion curve became steeper. Further studies are required to learn more about the cause of this effect. However, it is possible that the patch is interfering with the ability of the skin barrier to retain water in the skin; therefore, when the skin is occluded water rapidly builds up.

As the skin was measured 0 min, 30 min and four hours after the removal of the patches, it was possible to investigate how rapidly the properties of the skin returned to their untreated state. We found that the regions treated with the film-backed patches remained significantly more hydrated than the initial state of the skin after four hours, whereas the regions treated with woven-backed patches had begun to return to their initial state after four hours. We also measured patches with different percentages of propylene glycol. It appeared that the patches with 3% propylene glycol led to the largest increase in skin hydration. However, these differences between propylene glycol content were smaller than the changes between the two backing materials. To further explore the effect of different excipients, a larger scale study should be conducted using more subjects to obtain significant results.

The ability to quantify the hydrating effects of patches for transdermal drug delivery, and how long these effects last, is important when designing patches to deliver drugs at a fixed rate as the rate of drug absorption through the skin barrier is closely linked to skin hydration. If the skin hydration increases due to the application of the patch, then the rate of drug delivery could also change and risk leaving the therapeutic window. Equally, it is important that the rate of recovery of the skin following the removal of the patches is well understood, as this can influence how soon patches can be safely reapplied to the same area and how long a patch can be left on the skin. Excess hydration of the skin due to prolonged patch application can lead to skin irritation and, if a patch is reapplied to the same region, then the hydration levels will be different from untreated skin, meaning that the initial rate of drug delivery will also be affected. THz measurements of the skin can therefore provide important insights into the effects of patches on the distribution of water in the skin following the application of different types of patches. Unlike other approaches which have been used to test the skin, such as TEWL measurements, THz measurements can give a direct indication of the water content of skin and can be used to observe the dynamic response of skin to occlusion. Many other approaches to testing the effects of patches are performed on animals or on excised tissue [38] but these studies do not give the same insight to the response of living human skin to the application of the patches.

This study offers a proof of concept, showing that THz technology has promising potential in this field and could be used to help develop more complex transdermal drug patches by increasing understanding of how patches influence the skin. To develop this work further, larger scale studies should be performed with more participants and a wider range of patch types with different excipients. Additionally, patches containing active ingredients should be tested to explore what effects these may have, particularly in cases where there is the potential for skin irritation. This study used THz point scans meaning that each measurement only observed a single location within the region of interest. As imaging speeds increase for THz systems it would be beneficial to obtain THz images of regions treated with patches to explore the spatial uniformity of the responses to the patches, for example, if the change in the skin is different near the edges of the patch. Finally, further work should be done to develop a model that can be used to investigate the effect of patch application on the diffusivity of water within the skin, such as that performed by Sun et al. [26]. However, this would require more complex models, as the skin is often assumed to be made of water and a biological background; if drugs and excipients are entering the skin then the models would need to be adapted to account for this [39].

## 5. Conclusions

In this study we have used THz measurements of the skin to quantify the response of skin to the application of fully and partially occlusive transdermal drug patches. It was possible to observe the increase in water content caused by both patch types and that the skin treated with a film-backed patch remained at an increased hydration level four hours after the removal of the patch. It was also observed that the response of skin to occlusion by the imaging window altered depending on which patch had been applied, providing further insight into the effect of patches on the distribution of water in the skin. The results of this study suggest that THz measurements of the skin could be used when developing patches for transdermal drug delivery to understand the effect of different patch backings on the water content of skin and, therefore, the rate of drug delivery.

## Data Availability

The datasets used in this work are available from figshare with doi:10.6084/m9.figshare.16685293.
